# Molecular monitoring of the diversity of human pathogenic malaria species in blood donations on Bioko Island, Equatorial Guinea

**DOI:** 10.1186/s12936-019-2639-8

**Published:** 2019-01-15

**Authors:** Tobias Schindler, Tamy Robaina, Julian Sax, Jose Raso Bieri, Maximilian Mpina, Linda Gondwe, Ludmila Acuche, Guillermo Garcia, Carlos Cortes, Carl Maas, Claudia Daubenberger

**Affiliations:** 1 Department of Medical Parasitology and Infection Biology, Swiss Tropical and Public Health Institute, Basel, Switzerland; 2 University of Basel, Basel, Switzerland; 3 Malabo Blood Bank, Malabo, Equatorial Guinea; 4 Equatorial Guinea Malaria Vaccine Initiative, Malabo, Equatorial Guinea; 5 Bagamoyo Research and Training Centre, Ifakara Health Institute, Bagamoyo, United Republic of Tanzania; 6 Medical Care Development International, Silver Spring, USA; 7 Marathon EG Production Ltd, Malabo, Equatorial Guinea

**Keywords:** Transfusion-transmitted malaria, *P. falciparum*, *P. malariae*, *P. ovale*, qPCR

## Abstract

**Background:**

Malaria can be transmitted by blood transfusion from human to human and it is responsible for the majority of transfusion-transmitted infectious diseases worldwide. In sub-Saharan Africa, it had been estimated that almost a quarter of blood donations contain malaria parasites. Since rapid diagnostic tests and thick blood smear microscopy lack sensitivity for low density parasitaemia, particularly in asymptomatic adults, the most reliable method to assess the problem of transfusion-transmitted malaria are nucleic acid-based molecular approaches such as quantitative polymerase chain reaction. The study was undertaken to determine the prevalence of sub-microscopic malaria parasite infection among blood donors in Malabo, Equatorial Guinea.

**Methods:**

Between July and August 2017, a total of 200 individual blood samples from blood donors at the Malabo Blood Bank were collected and screened by rapid diagnostic tests and thick blood smear microscopy. Retrospectively, the same samples were analysed for the presence of undetected, low-density malaria parasites using quantitative polymerase chain reaction.

**Results:**

In comparison to 6.5% (13/200) by rapid diagnostic test and 2.0% (4/200) by microscopy, the proportion of *Plasmodium falciparum* positive blood donations analysed by quantitative polymerase chain reaction was significantly higher (26%, 52/200). Densities of *P. falciparum* positive blood donations were ranging from 0.06 to 3707.0 parasites/µL with 79.6% below 100 parasites/µL and therefore not detectable by non-molecular malaria diagnostic tests. qPCR based species identification revealed that *P. falciparum* was the dominating species responsible for 88.1% (52/59) of positive blood donations, followed by *Plasmodium malariae* (15.3%, 9/59) and *Plasmodium ovale* (3.4%, 2/59).

**Conclusions:**

This study confirms that in malaria endemic settings, sub-patent malaria infections among blood donors are prevalent. In blood collected from healthy donors living in Malabo, *P. falciparum*, *P. malariae* and *P. ovale* parasites were identified. Currently widely used malaria diagnostic tools have missed more than 75% of *P. falciparum* containing blood donations, demonstrating the value of quantitative polymerase chain reaction to reliably detect low density *P. falciparum* infections. Since the availability of molecular diagnostic methods in malaria endemic countries is still limited, the blood recipients living in malaria endemic countries should be treated following WHO recommendations.

**Electronic supplementary material:**

The online version of this article (10.1186/s12936-019-2639-8) contains supplementary material, which is available to authorized users.

## Background

Malaria is a vector-borne parasitic tropical disease found in 55 countries globally [1]. Six *Plasmodium* species are known to infect humans including *Plasmodium falciparum*, *Plasmodium vivax*, *Plasmodium malariae*, *Plasmodium ovale curtisi*, *Plasmodium ovale wallikeri* and *Plasmodium knowlesi* [2]. Malaria is transmitted to the human host through the bite of a female *Anopheles* mosquito when the sporozoite stage is inoculated during the feeding. The parasite undergoes a pre-erythrocytic liver stage reproduction that is clinically silent and lasts 1 to 2 weeks before the release of merozoites into the bloodstream. These merozoites rapidly infect red blood cells, starting the asexual blood stage whereby the parasite undergoes cycles of asexual replication that include the ring, trophozoite, and schizont stages leading to regular fever episodes [2]. In adult human populations living in malaria endemic countries, the prevalence of sub-patent malaria infections is up to 80% [3] with certain parasite clones surviving in the human host more than 300 days [4].

Diagnosis of malaria is performed in blood samples collected from potentially infected humans. The gold standard for malaria diagnosis remains light microscopy of stained blood smears. Thick blood smears provide sensitivity and thin smears allow the identification of different malaria species and the quantitation of infection [5]. Rapid diagnostic tests (RDTs) are widely used since they offer less dependence on the availability of laboratory infrastructure, and can also be employed by inexperienced health workers [6]. RDTs are often based on parasite-derived histidine-rich protein 2 (PfHRP2) for sensitive and specific detection of *P. falciparum,* and lactate dehydrogenase enzyme (LDH) or aldolase for a *Pan*-*Plasmodium* detection of all human infective malaria species [7]. The lower limit of detection (LOD) for microscopy is between 50 and 500 parasites/µL depending on the expertise of the microscopist [5] and 100 parasite/µL for the PfHRP2 based *P. falciparum* RDTs [8]. Differential diagnosis of *P. ovale*, *P. malariae*, *P. vivax* and *P. knowlesi* is hampered by the low density blood stage infections and the difficulty in distinguishing these species based on morphology of trophozoites using microscopy [9].

Nucleic acid amplification technologies (NATs) are more expensive and require advanced laboratory infrastructure but provide much better sensitivity. The parasites’ multi-copy 18S small subunit ribosomal deoxyribonucleic acid (rDNA) and/or its highly expressed ribonucleic acid (rRNA) is the most frequently used biomarker for NATs [10]. Different methods for 18S rDNA/rRNA detection are currently in use with a great variation in sensitivity. Qualitative methods targeting 18S rDNA, such as nested polymerase chain reaction (PCR) [11] and LAMP [12], reach LODs of 0.1–10 parasites/µL. The use of quantitative PCR (qPCR) [13], does not only allow absolute quantification of infections but also lowers the LOD to less than 1 parasite/µL. The use of Reverse Transcription quantitative PCR (RT-qPCR), amplifying total nucleic acids (RNA and DNA) of the 18S genes [14–16], and qPCR assays targeting alternative multi-copy genomic sequences [17] further increase sensitivities of NATs. Apart from increased sensitivity of NATs, molecular detection using PCR has improved species discrimination compared to either microscopy or RDTs [18].

Screening for transfusion transmitted infections should follow the World Health Organization (WHO) recommendations which include searching for chronic infectious diseases including HIV-1, HIV-2, Hepatitis B, Hepatitis C and *Treponema pallidum* (syphillis) [19]. Based on the local epidemiological infectious disease situation, the WHO recommends that blood donations should also be screened for malaria [19, 20]. Malaria can be efficiently transmitted by blood transfusion from human to human and is undoubtedly responsible for the majority of transfusion transmitted diseases in the world [21]. The rate of transfusion-transmitted malaria (TTM) in malaria endemic sub-Saharan regions is estimated between 14 and 28% [22]. Several reports describe that *P. falciparum* [22], *P. vivax* [23], *P. malariae* [24], *P. knowlesi* [25] and *P. ovale* [26] can be transmitted either through blood donations or solid organ transplantations.

Malaria species circulating in Equatorial Guinea include *P. falciparum*, *P. malariae*, *P. ovale* and *P. vivax* [27–29]. The Bioko Island Malaria Control Project (BIMCP) started in 2004 with the aim to reduce malaria transmission and to control the burden of disease and has received substantial funding from the Government of Equatorial Guinea and private donors including Marathon EG Production Limited, Noble Energy, and Atlantic Methanol Production Company. The malaria control strategy of the BIMCP rests on vigorous vector control measures, effective case management, prevention of malaria during pregnancy, behavioural change communications and regular monitoring and evaluation [30]. The BIMCP has had significant success in reducing *P. falciparum* parasite prevalence in the 2–14 years old children and adolescent from 45% (95% CI 40–50%) in 2004 (baseline) to 12.1% (95% CI 11.2–13.3%) in 2016 based on annual Malaria Indicator Survey (MIS) data [30].

Since RDTs and microscopy lack sensitivity for low-level parasitaemia, particularly in asymptomatic adults, the most reliable method to assess the problem of TTM are NATs such as qPCR. This study has set out to analyse a selection of 200 blood samples from the blood bank in Malabo using qPCR to identify sub-patent *P. falciparum* infections that cannot be detected by commonly used RDTs and microscopy. In addition, the presence of other TTM causing *Plasmodium* species was analysed using a newly developed single tube multiplex qPCR assay.

## Methods

### Study site and participant recruitment

The study was conducted at the Central Blood Bank in Malabo on Bioko Island. Bioko Island, located in West-central Africa and home of Equatorial Guinea’s capital city Malabo, is 32 km from the coast of Cameroon. The approximately 250,000 people, who mainly reside in Malabo are at risk of malaria year-round [30]. Adults willing to donate blood and who are Hepatitis B, Hepatitis C, and HIV negative were eligible for blood donation. Microscopy and RDT (CareStart™ Malaria HRP2/pLDH Pf/PAN Combo) were used for malaria screening at the blood bank and the results were kept at the blood bank until the end of qPCR analysis. The Malabo Central Blood Bank processes around 100 donors per month; the majority being donors assisting friends and families during emergency situations, and the remainder are donors that voluntarily donate blood on a regular basis. The blood bank is run by a public–private cooperation between the Ministry of Health and Social Welfare, the University of Valencia Hospital, and funded by the AGEM-GUINEA company.

### Sample collection for molecular diagnostics

Between July and August 2017, a total of 200 individual blood donations from routine visitors were selected to conduct this study. 1 mL of whole blood was collected in EDTA tubes as part of the usual blood donation procedures in the blood bank. The blood was immediately used for microscopy and RDT, with the remaining blood immediately stored at − 20 °C. The samples were transported in a cooling box to the laboratory of the Equatorial Guinea Malaria Vaccine Initiative (EGMVI) in Malabo. The laboratory infrastructure of the EGMVI, located on the premises of the La Paz Hospital in Malabo, which conducted the first clinical trial in the history of the country [31], was used for qPCR analysis of the blood donations.

The 200 blood samples, from healthy, Hepatitis B, Hepatitis C, and HIV-1 and HIV-2 negative blood donors were analysed for the presence of possibly undetected, low-level malaria parasites using high-sensitive qPCR assays. DNA extraction was done manually from 180 µL whole blood using Quick-DNA Miniprep kits (Zymo Research, Irvine, USA) following manufactures’ guidelines and DNA was eluted with 50 µL Elution Buffer. DNA samples were kept at − 20 °C prior to qPCR analysis.

### qPCR for *Plasmodium* screening and quantification (PlasQ assay)

*Plasmodium* spp. and *P. falciparum* parasites were quantified using the PlasQ assay. This assay consists of two independent *Plasmodium* targets combined in a multiplex assay. The *Pan*-*Plasmodium* 18S rDNA sequence (Pspp18S) [13, 32], and the *P. falciparum*-specific acidic terminal sequence of the *var* genes (PfvarATS) [17] were used for detection and quantification of parasites. The human RNaseP (HsRNaseP) gene served as an internal control to assess the quality of DNA extraction and qPCR amplification.

Amplification and qPCR measurements were performed using the Bio-Rad CFX96 Real-Time PCR System (Bio-Rad Laboratories, California, USA). The thermal profile used for PlasQ qPCR is as follows: 60 s at 95 °C; 45 cycles of 15 s at 95 °C and 45 s at 57 °C. Each reaction contained 2 µL DNA and 8 µL reaction master mix containing 1× Luna Universal Probe qPCR Master Mix (New England Biolabs, Ipswich, USA) and 1× PlasQ Primer Mix (Additional file 1). All qPCR assays were run in duplicates with appropriate controls including Non-Template Control [NTC] and *P. falciparum* 3D7 DNA as positive control [PC]. The mean Cq of the two replicates was reported and in case of one qPCR replicate interpreted as malaria positive and the other replicate negative, then the assay had to be repeated to arrive at an unequivocal result.

Using the first WHO International Standard for *P. falciparum* DNA Nucleic Acid Amplification Techniques (NIBSC code: 04/176), a serial dilution ranging from 0.05 to 10,000 parasites/µL was prepared and used for quantification of *P. falciparum*. The WHO standards were run as duplicates in two out of the total 13 qPCR runs conducted.

### qPCR for *Plasmodium* species identification (PlasID assay)

Several published [17, 33–37] and unpublished qPCR assays, detecting *P. falciparum*, *P. malariae*, *P. ovale*, *P. vivax* and *P. knowlesi* were evaluated and the best performing primer and probes for each *Plasmodium* species were combined into a new multiplex assay (PlasID). The primers and probe combinations used for this novel pentaplex malaria qPCR assay is provided in Additional file 1.

Amplification and qPCR measurements were performed using the Bio-Rad CFX96 Real-Time PCR System (Bio-Rad Laboratories, California, USA). The thermal profile used for the PlasID qPCR is as follows: 15 min at 95 °C; 45 cycles of 15 s at 95 °C and 60 s at 55 °C. Each reaction contained 2 µL DNA and 8 µL reaction master mix containing 1× HOT FIREPol^®^ Probe qPCR Mix Plus (Solis Bio Dyne, Tartu, Estonia) and 1× PlasID Primer Mix (Additional file 1). All qPCR assays were run in duplicates with appropriate controls including Non-Template Control [NTC] and DNA of six *Plasmodium* species as positive controls [PC]. DNA controls for *P. falciparum*, *P. knowlesi*, *P. malariae*, *P. vivax*, *P. ovale curtisi* and *P. ovale wallikeri* were used to evaluate species specificity of the PlasID assay. The positive controls were reconfirmed by two commercial qPCR assay (GeneFinder™ Malaria RealAmp *Pf/Pv/Pk* and *Pf/Pm/Po*). Samples with a Cq value less than 45 for any of the five *Plasmodium* targets were considered positive for the corresponding *Plasmodium* species.

### Data management and analysis

Geneious version 8.1.9 (Biomatters Ltd, Auckland, New Zealand) was used for sequence alignments and oligonucleotide designs. Cq values were obtained from the Bio-Rad CFX96 Manager 3.1 software (Bio-Rad Laboratories, California, USA) and transferred to a Microsoft Access based in-house database designed for storage and analysis of qPCR data. Cq values were transformed to parasite densities and linked to patient’s metadata collected at the blood bank. Categorical variables were compared by Fisher’s exact test and continuous variables by Mann–Whitney using GraphPad Prism version 7.00 for Windows (GraphPad Software, La Jolla, USA). P values < 0.05 were considered to be significant.

## Results

### Development and implementation of multiplex qPCR assays for malaria screening

**Fig. 1:**
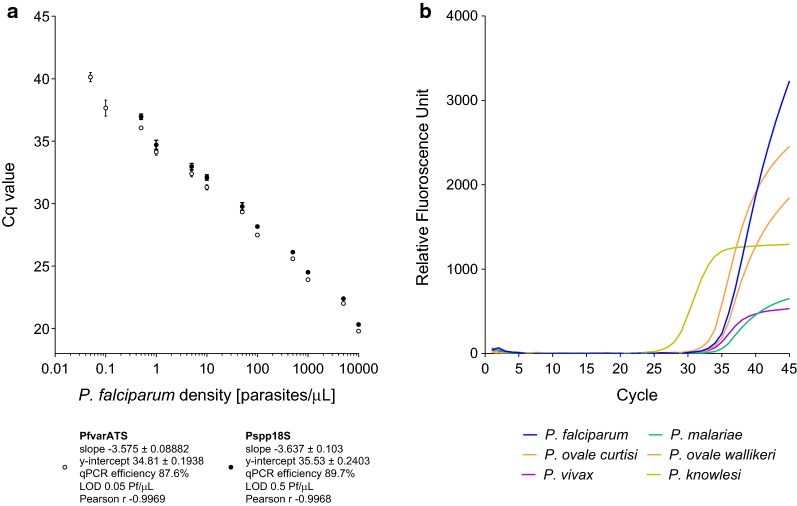
Analytical performance of PlasQ (**a**) and PlasID (**b**) assay. **a** Correlation of *P. falciparum* standards and the Cq values for both targets, Pspp18S (black circles) and PfvarATS (white circles) of the PlasQ assay. Based on these quadruple replicates of the WHO standards, LODs and qPCR efficiencies were calculated. **b** The ability of the PlasID assay identifying five different malaria species is shown by a representative, composite amplification plot. DNA from the six *Plasmodium* species were analysed in separate tubes during the same qPCR experiment

Two independent qPCR assays were systematically used for the screening and identification of malaria parasites. The use of two consecutive qPCR assays maximizes the amount of information generated by the analysis. The first assay (PlasQ), applied to all samples, was designed to identify and quantify *P. falciparum* and/or non-*falciparum* species. Additionally, the internal control serves as a general control for quality and performance of the DNA extraction and qPCR reaction. The performance of the PlasQ assay is shown in Fig. 1a. For both targets, PfvarATS and Pspp18S, the Cq values for different parasite densities, ranging from 0.05 to 10,000 parasites/µL were obtained (Pearson r − 0.9969 and − 0.9968, respectively). Similar qPCR efficiencies for both targets were observed (87.6% and 89.7%). The PfvarATS target did detect two additional samples carrying low parasite densities (0.1 and 0.05 parasites/µL) resulting in 10 times lower LOD compared to the Pspp18S target. The second assay (PlasID), which is applied to all *Plasmodium* spp. positive samples, was designed for rapid identification of five different malaria causing *Plasmodium* species. Performance of the PlasID assay was assessed using well-defined clinical samples as references and this novel assay’s ability of identifying *P. falciparum*, *P. malariae*, *P. ovale curtisi*, *P. ovale wallikeri*, *P. vivax* and *P. knowlesi* is demonstrated in Fig. 1b.

### Baseline characteristics of blood donors attended Malabo Central Blood Bank

Blood donor characteristics are given in Table 1. All donors were Equato-Guineans living in Malabo on Bioko Island except two persons, one originating from neighbouring Douala, Cameroon and the other from the United States. The majority were male (88.5%) with a median age of 32 years, ranging from 18 to 57 years. Seventy-seven donations were from voluntary blood donors and 123 donations were from replacement donors, who donate blood when required by a member of the patient’s family or community (Table 1). Except for the blood pressure, no significant difference in relation to gender, age, weight, pulse and anaemia status between these two donor groups were observed.

**Table 1:** Comparison of health characteristics between voluntary and replacement donors

	Voluntary donors	Replacement donors	P values^a^
Number of donors	77	123	–
% male	84.4% (65/77)	91.1% (112/123)	0.175
Age in years^b^	32 [19–55]	32 [18–57]	0.888
Weight in kg^b^	73.9 [51–116]	72.0 [52–116.6]	0.593
Blood pressure in mmHg^b^	126.7 [97.7–169.0]	130.8 [103.6–198.9]	0.035
Pulse in bpm^b^	74 [48–101]	73 [51–102]	0.788
Haemoglobin in g/dL^b^	14.6 [12.2–19.6]	14.7 [6.4–18.0]	0.808

^a^Variables were compared by Fisher’s exact test for categorical variables or Mann–Whitney test for continuous variables

^b^Values expressed as medians with ranges

### Prevalence of malaria positive blood donations

**Fig. 2:**
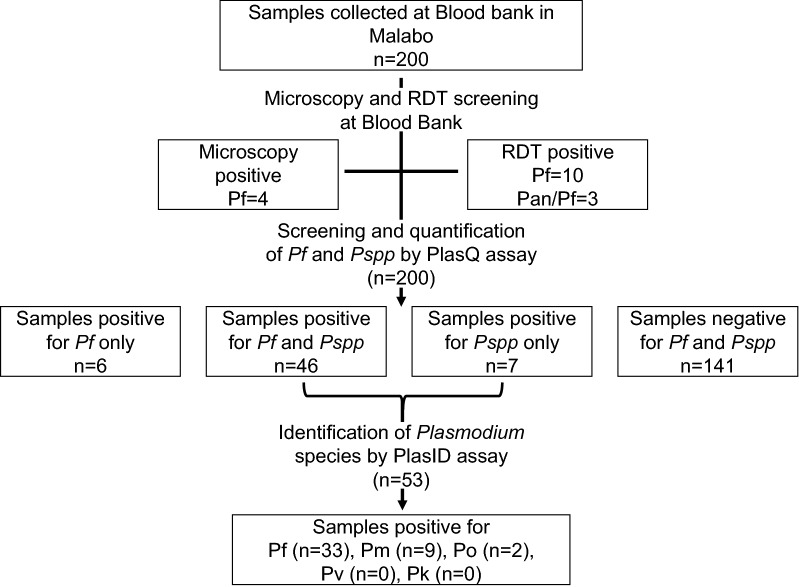
Overview of blood sample analysis. Blood donations were systematically screened for the presence of malaria parasites by microscopy, RDT and qPCR assays

The flow diagram depicting the sequential malaria diagnostic tools applied to the blood donations is summarized in Fig. 2. In the Malabo blood bank, all samples were screened using thick blood smear microscopy and RDT. Microscopy identified four *P. falciparum* positive donations, while RDTs detected 13 malaria positive donations. Three donations were positive for both antigens, the *P. falciparum* specific HRP2 and the *Plasmodium* spp. LDH antigens. The remaining ten donations were positive for HRP2 only. Microscopy and RDT positive blood donations were not considered for donation and were included blinded into this study sample collection on purpose to test the performance of the PlasQ assay.

Upon arrival at the EGMVI laboratory all samples were screened with the first multiplex qPCR assay (PlasQ), targeting *P. falciparum* (PfvarATS), *Plasmodium* spp. (Pspp18S) and the human RNAseP gene (as an internal control). All 200 samples amplified the internal control with Cq < 28 and were therefore eligible for further analysis. Over 70% (n = 141) of blood donations were negative for any malaria species. Forty-six donations were positive for both *Plasmodium* targets, while six and seven donations were positive only for *P. falciparum* or *Plasmodium* spp., respectively. All samples (n = 53) with a positive amplification of the Pspp18S target were analysed with the species identification assay (PlasID). Apart from confirming *P. falciparum* (n = 33) cases, we also found cases of *P. malariae* (n = 9) and *P. ovale* (n = 2). Amongst the 200 blood samples analysed, no *P. vivax* or *P. knowlesi* was detected.

The assay identified *P. malariae* or *P. ovale* in all seven samples which were positive for *Plasmodium* spp. and negative for *P. falciparum*, highlighting the assays ability to identify non-*falciparum* species. Compared to the PlasQ assay, a reduced sensitivity (71.7%, 33/46) for *P. falciparum* detection using the PlasID assay was observed. The 13 *P. falciparum* positive samples missed by the PlasID assay had moderate parasitaemia (median of 36.9 with IQR of 1.3–380.0 parasites/µL) and the five samples with the highest parasitaemia were positive by RDT. None of these samples were positive for any other non-*P. falciparum* species.

An additional 29 malaria negative blood samples were also run with the PlasID assay to test specificity of the assay. No false positive *Plasmodium* spp. cases were detected, resulting in 100% specificity.

Notably, none of the non-*falciparum* species had been identified by microscopy or RDT. Pan-positive RDTs, which detected the *Plasmodium* spp. LDH, were rather associated with higher *P. falciparum* density than with the detection of non-*falciparum* malaria parasite species. The three HRP2 and LDH positive RDTs had a geometric mean of 916.3 parasites/µL (range 244.1–3707.0) while the nine HRP2 only positive RDTs had a geometric mean of 106.0 parasites/µL (range 11.2–543.3) (Table 2).

**Table 2:** Parasitaemia of blood donations detected with different diagnostic methods

Diagnostic tool	Median/IQR (parasites/µL)^a^	Geometric mean (parasites/µL)^b^	Range (parasites/µL)
qPCR-PfvarATS	4.6 [0.8–49.0]	5.6 [2.6–12.1]	0.06–3707.0
qPCR-Pspp18S	7.8 [1.3–65.1]	9.5 [4.5–19.8]	0.07–3707.0
RDT combined^c^	134.6 [65.1–536.5]	181.7 [68.98–478.7]	11.16–3707.0
HRP2-RDT^d^	112.5 [52.2–331.2]	106.0 [42.4–265.1]	11.16–3707.0
HRP2/LDH-RDT^e^	850.4 [244.1–3707.0]	916.3 [31.11–26990.0]	244.1–3707.0
Microscopy	380.1 [145.4–2909.0]	478.7 [44.03–5205.0]	112.5–3707.0

^a^Quartiles (Median, 25th and 75th percentile)

^b^Geometric mean and 95% confidence interval

^c^Pf- and Pf/Pan-RDT results combined

^d^Pf-RDT results only

^e^Pf/Pan-RDT results only

In summary, 59 blood donations were positive for at least one malaria species (29.5%). *Plasmodium falciparum* was the dominating species responsible for 88.1% of positive blood donations, followed by *P. malariae* (15.3%) and *P. ovale* (3.4%). Mixed species infections were found in 6.8% (4/59) of the malaria positive blood donations. One blood donation carried a *P. malariae* and *P. ovale* co-infection and three donations contained a combination of *P. falciparum* and *P. malariae*. The observed co-infection proportion between *P. falciparum* and *P. malariae* was slightly higher than expected (1.5% versus 1.2%, P = 0.037).

### *Plasmodium falciparum* densities in malaria positive blood donations

**Fig. 3:**
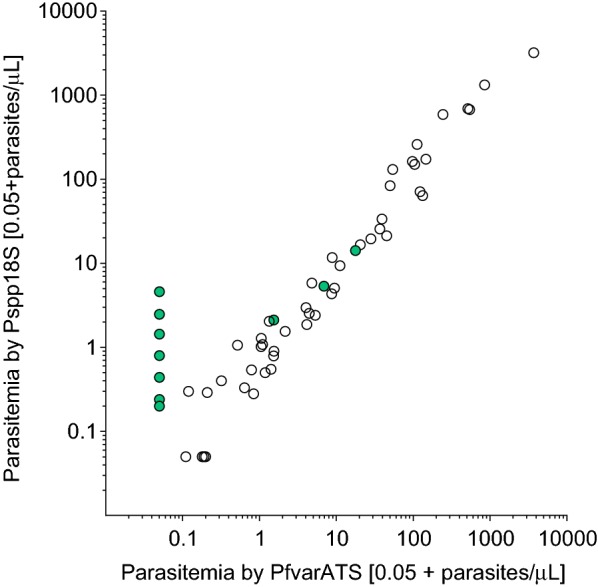
Parasite densities of positive blood donations obtained from qPCR assays. Strong correlation between the two targets is observed (Spearman r 0.894). The green dots highlight samples containing non-*falciparum* malaria species. An offset of 0.05 parasites/µL was used

Data obtained from the PlasQ assay was used to quantify *P. falciparum* positive blood donations. Parasite densities calculated based on both targets show a high correlation (Spearman r 0.894, Fig. 3). Identified non-*falciparum* species are indicated with green dots. Quantification based on the Pspp18S assay revealed, that the parasitaemia of these non-*falciparum* species is around or below 10 parasites/µL which was supported by the high Cq values obtained from the PlasID assay. The geometric mean Cq value of the nine *P. malariae* positive samples was 36.9 (range 35.3–39.0) and the two positive *P. ovale* samples had Cq values of 37.9 and 39.8.

**Fig. 4:**
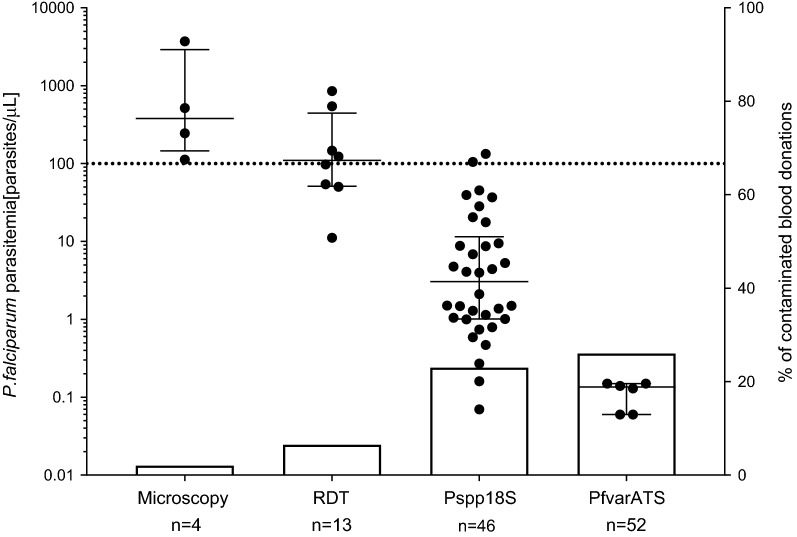
Association between parasitaemia and sensitivity of diagnostic methods applied in this study. Median and interquartile ranges of non-cumulative *P. falciparum* parasitaemia are grouped according to different diagnostic tests used in this study (left y-axis, scatter plot). The dashed line at 100 parasites/µL represents the widely accepted LOD for RDTs. Proportional rates of positive blood donations are represented as bars (right y-axis)

A strong association between the diagnostic tests and the parasitaemia in *P. falciparum* positive blood donations was observed. Non-cumulative parasite density data grouped by diagnostic test is shown in Fig. 4 (scatter plot, left y-axis).

In Table 2, the parasite densities grouped by diagnostic tool are summarized. Densities of *P. falciparum* positive blood donations ranged from 0.06 to 3707.0 parasites/µL with a median of 4.6 parasites/µL (IQR 0.8–49.0), which is lower than the LOD for conventional diagnostic tests. Only samples with the highest parasitaemia were detectable by microscopy (381.1 [145.4–2909.0] parasites/µL) and RDTs (134.6 [65.1–536.5] parasites/µL), while the two qPCR assays detected additional infections with lower parasite densities. The median parasitaemia was 7.8 parasites/µL (IQR 1.3–65.1) for the Pspp18S target and 4.6 parasites/µL (IQR 0.8–49.0) for the PfvarATS target (Table 2).

### Performance of diagnostic methods for *P. falciparum* detection in blood donations

In total, 48 previously undetected *P. falciparum* infections were identified by conducting the varATS based qPCR analysis compared to microscopy, increasing the proportion of infected blood donations from 2.0 to 26.0%. The detection rate for RDTs and 18S based qPCR were 6.5% and 23.0%, respectively (Fig. 4, bar plot, right y-axis). This increased detection rate of the qPCR assays is also reflected in analytical sensitivities of the diagnostic tests. Using the PfvarATS qPCR results as the gold standard for *P. falciparum* detection, Pspp18S qPCR (88.5%), RDT (23.1%) and microscopy (7.7%) have all reduced sensitivities. Specificities were for all tests above 95%. One RDT positive sample could not be confirmed by qPCR, while the specificity for Pspp18S was reduced because of non-*falciparum* malaria parasite species, which are also detected (Table 3).

**Table 3:** Analytical comparison of methods deployed for *P. falciparum* detection

Diagnostic tool	Sens^a^	Spec^b^	PPV^c^	NPV^d^
qPCR-PfvarATS	*Gold standard*			
qPCR-Pspp18S	88.5% [76.6–95.7]	95.3% [90.5–98.1]^e^	86.8% [76.0–93.2]^e^	95.9% [91.7–98.0]
RDT	23.1% [12.5–36.8]	99.3% [96.3–99.9]	92.3% [61.5–100.0]	79.0% [76.0–81.0]
Microscopy	7.7% [2.1–18.5]	100.0% [97.54–100]	100% –	75.5% [74.0–76.9]

^a^Sensitivity and 95% confidence interval

^b^Specificity and 95% confidence interval

^c^Positive predictive value and 95% confidence interval

^d^Negative predictive value and 95% confidence interval

^e^Specificity and PPV of Pspp18S qPCR assay is reduced because non-*falciparum Plasmodium* species are also detected

### Identification of risk factors for malaria positivity

Malaria positive and negative donors were stratified according to the differences in the questionnaire that all blood donors must fill before proceeding to blood donation (Additional file 2). The major risk factor for being a malaria positive blood donor was reporting fever or malaria during the past 3 weeks (Additional file 2). When comparing the anthropometric measurements between malaria positive and negative donors, marginal differences in age and weight become apparent (Table 4). An increased rate of positive blood donations in replacement donors (29.3%) compared to voluntary donors (20.8%) was observed, as well as an increased parasitaemia among replacement donors (8.9 versus 2.1 parasites/µL). However, these differences were not statistically significant.

**Table 4:** Anthropometric differences in *P. falciparum* positive versus *P. falciparum* negative blood donors

	Negative donors	Positive donors	P values^a^
Number of donors	148	52	–
% male	86.5% (128/148)	94.2% (49/52)	0.205
Age in years^b^	33 [18–57]	29 [20–55]	0.013
Weight in kg^b^	74.6 [51–116.6]	69.2 [53–110]	0.025
Blood pressure in mmHg^b^	128.7 [97.7–198.9]	128.3 [100.6–156.9]	0.326
Pulse in bpm^b^	74 [48–102]	72 [51–96]	0.519
Haemoglobin in g/dL^b^	14.6 [6.4–19.6]	14.7 [11.6–16.5]	0.319

^a^Fisher’s exact test for categorical variables or Mann–Whitney test for continuous variables was applied

^b^Values expressed as medians with ranges

## Discussion

This study, conducted in Equatorial Guinea, extends previous observations of presence of TTM in sub-Saharan Africa, and demonstrates the value of NAT approaches to reliably detect sub-patent *P. falciparum* and non-*falciparum* malaria parasites including *P. malariae* and *P. ovale.* Apart from *P. falciparum*, other malaria causing species such as *P. vivax*, *P. malariae*, and *P. ovale* have been described to circulate on Bioko Island—similar to neighbouring West African countries [27, 28]. Transfusion of any of these malaria species during blood donations have the potential to cause severe disease in recipients. In sub-Saharan Africa, those most at risk are anaemic infants and children, pregnant women, or women who suffer from high blood losses while giving birth [25, 38, 39]. Although, there is a high frequency of TTM in malaria endemic regions, the semi-immunity of recipients may largely control parasite replication and therefore clinical outcome [40].

In malaria non-endemic countries, TTM needs also be taken into account to ensure safe blood donations [41]. Here, blood the recipients are mainly malaria naïve and the immunological control of potentially transfused parasites is non-existent resulting in in-creased risk of severe malaria disease. *P. falciparum* parasites have been found to survive in blood donations for up to 14 days limiting the potential to neutralize malaria during blood donation processing and storage [42].

This study demonstrates the challenges blood banks in malaria endemic countries encounter. The blood donors at the Malabo blood bank were mainly male adults within an age range of 18–57 years. Because of their pre-existing immunity, blood donors of that age tend to have low levels of parasites without exhibiting clinical symptoms. Ninety-seven percent (97.0%) reported to be in good health at the time of blood donations. Apart from self-reported malaria and/or fever episodes within the last 3 weeks, no other risk factor had been identified based on the provided questionnaire. Since only about 3% (6/198) had reported to have malaria and/or fever episodes, the use of this risk factor to pre-select blood donors would not be very efficient. Deferral of about a quarter of blood donors that are sub-patent malaria carriers in a region where blood supply shortage is eminent could be even more damaging to public health.

Conventional diagnostic tests are important tools, since they are affordable and can be applied immediately at blood banks, but lack in sensitivity limits their usefulness. Microscopy is still an important tool for malaria detection since it allows for species identification and quantification. The detection limit of an expert microscopy is close to 20–50 parasites/µL, but for non-experienced microscopists, it is up to tenfold higher (i.e. 500 parasites/µL) [5]. Currently available RDTs are based on detection of circulating, parasite derived HRP2, aldolase or lactate dehydrogenase in serum or whole blood samples. These RDTs have the ability to detect *Plasmodium* spp. infections with a LOD between 100 and 1000 parasites/µL which is significantly higher when compared to NATs [43]. In addition, there is currently no single RDT available that could distinguish all five malaria species infections posing a clear limitation to comprehensive malaria infection status monitoring [44, 45]. Infections with *P. malariae* [46], *P. ovale* [47] and *P. vivax* [48] are usually characterized by low parasitaemia levels, rendering these infections largely undetectable by using currently available RDT technologies [49].

In this study, more than three-quarter of *P. falciparum* and all non-*falciparum* infections were missed by the conventional diagnostic tools. A suitable screening method for malaria detection in blood units must have high sensitivity, high negative predictive value, must include all *Plasmodium* species, and should be cost efficient. Such a test must enable a reduction of TTM risk, as well as that of falsely deferred blood donors found to have false positive results.

The PlasQ malaria screening assay applied in this study meets some of these requirements. With a LOD of 0.05 parasites/µL, PlasQ has high sensitivity for *P. falciparum* and includes all other relevant *Plasmodium* species. This assay was extensively used during controlled human malaria infections in Tanzanian and Equatorial Guinean volunteers and no issues with specificity were observed (unpublished). As a limitation, qPCR analysis requires an advanced laboratory infrastructure, trained personnel and the costs for reagents and consumables for this assay is about USD 5, which is higher than the costs for RDT or microscopy-based malaria diagnosis.

This study was used to obtain more information about non-*falciparum* malaria species circulating in Malabo. Therefore a novel multiplex qPCR assay for parallel malaria parasite identification was developed. Using the PlasID assay, the main five human infectious malaria species can be detected qualitatively in a multiplex assay rendering it highly cost efficient since only one DNA extraction and qPCR reaction needs to be run. This novel malaria species identification assay should be used in combination with the PlasQ assay, based on the finding that in the PlasID assay, a reduced sensitivity for *P. falciparum* is observed. Further development of diagnostic assays for the detection of *P. malariae* and *P. ovale*, should include the ability of species-specific quantification. Estimations based on Pspp18S target of the PlasQ assay, indicate that *P. malariae* and *P. ovale* single infections had a geometric mean density of about 0.6 parasites/µL, which is more than 14 times lower than *P. falciparum* single infections (8.8 parasites/µL).

Educating the general public in malaria endemic regions via TV, radio, and pamphlet about the existence of TTM and its potential health impact might help to raise the awareness of this malaria transmission route, both in potential blood donors and medical and nursing staff involved in blood transfusions [50]. This study confirms prior reports that replacement blood donors are particularly prone to harbour sub-patent malaria parasites [51]; hence this sub-population of donors are optimal for such awareness campaigns regarding potential TTM when donating blood for a family or friend. In addition to becoming aware of the potential TTM, medical and nursing staff should also use anti-malarial interventions when the potential for TTM is high and the malaria diagnostic infrastructure is limited. Ultimately, if the goal of malaria elimination is pursued vigorously, tackling TTM in sub-Saharan Africa needs to be included into the malaria elimination agenda.

## Conclusions

More than one quarter of blood donations from healthy donors living in Malabo were infected with malaria parasites. Three species, including *P. falciparum*, *P. malariae* and *P. ovale* were detected using two different qPCR assays. The majority of these infections were not identified by the currently widely used malaria diagnostic tools such as RDTs and microscopy. Since the availability of molecular diagnostic methods in malaria endemic countries is still limited, the blood recipients living in malaria endemic countries should be treated following WHO recommendations.

## Additional files

**Additional file 1.** Oligos used in this study.

**Additional file 2.** Identification of risk factors associated with a malaria positive blood donation.

## Abbreviations

RDT: rapid diagnostic test
HRP2: histidine rich protein 2
LDH: lactate dehydrogenase
LOD: limit of detection
NAT: nucleic acid amplification technology
qPCR: quantitative polymerase chain reaction
TTM: transfusion-transmitted malaria
EGMVI: Equatorial Guinea Malaria Vaccine Initiative
BIMCP: Bioko Island Malaria Control Project
Pspp18S: *Pan*-*Plasmodium* 18S rDNA sequence
PfvarATS: *P. falciparum*-specific acidic terminal sequence of the *var* genes
