# Supplementary material for: Molecular monitoring of the diversity of human pathogenic malaria species in blood donations on Bioko Island, Equatorial Guinea
Source: Malar J. 2019 Jan 15;18:9. doi: 10.1186/s12936-019-2639-8 (PMC6332537; doi:10.1186/s12936-019-2639-8)
Supplement: Supplementary file 1 — Additional file 1. Oligos used in this study. [file 12936_2019_2639_MOESM1_ESM.docx]

Additional file 1. Oligos used in this study.

| **Oligo name** | **Species specificity** | **Target region** | **Oligo sequence** | **Oligo modification [5’-3’]** | **Concentration 5x Oligo Mix** | **Adapted from** |
| --- | --- | --- | --- | --- | --- | --- |
|  |  |  |  |  |  |  |
| **a) PlasQ assay** |  |  |  |  |  |  |
|  |  |  |  |  |  |  |
| Pspp18S fwd | *Plasmodium spp* | 18S rDNA | GCT CTT TCT TGA TTT CTT GGA TG | - | 2 µM | Kamau et al, 2013 [13] |
| Pspp18S rev | *Plasmodium spp* | 18S rDNA | AGC AGG TTA AGA TCT CG TTC G | - | 2 µM | Kamau et al, 2013 [13] |
| Pspp18S probe | *Plasmodium spp* | 18S rDNA | ATG GCC GTT TTT AGT TCG TG | Cy5-BHQ2 | 1 µM | Kamau et al, 2013 [13] |
| HsRNaseP fwd | *H. sapiens* | Rnasep gene | AGA TTT GGA CCT GCG AGC G | - | 1 µM | Kamau et al, 2013 [13] |
| HsRNaseP rev | *H. sapiens* | Rnasep gene | GAG CGG CTG TCT CCA CAA GT | - | 1 µM | Kamau et al, 2013 [13] |
| HsRNaseP probe | *H. sapiens* | Rnasep gene | TTC TGA CCT GAA GGC TCT GCG CG | YakimaYellow-BHQ1 | 0.5 µM | Kamau et al, 2013 [13] |
| PfvarATS fwd | *P. falciparum* | varATS | CCC ATA CAC AAC CAA YTG GA | - | 1 µM | Hofmann et al, 2015 [17] |
| PfvarATS rev | *P. falciparum* | varATS | TTC GCA CAT ATC TCT ATG TCT ATC T | - | 1 µM | Hofmann et al, 2015 [17] |
| PfvarATS probe | *P. falciparum* | varATS | TRT TCC ATA AAT GGT | FAM-NFQ/MGB | 0.5 µM | Hofmann et al, 2015 [17] |
|  |  |  |  |  |  |  |
| **b) PlasID assay** |  |  |  |  |  |  |
|  |  |  |  |  |  |  |
| PfvarATS fwd | *P. falciparum* | varATS | CCC ATA CAC AAC CAA YTG GA | - | 2 µM | Hofmann et al, 2015 [17] |
| PfvarATS rev | *P. falciparum* | varATS | TTC GCA CAT ATC TCT ATG TCT ATC T | - | 2 µM | Hofmann et al, 2015 [17] |
| PfvarATS probe | *P. falciparum* | varATS | TRT TCC ATA AAT GGT | FAM-NFQ/MGB | 1.25 µM | Hofmann et al, 2015 [17] |
| PmPlasp4 fwd | *P. malariae* | Plasp4 gene | CCA ACA ATA CAT ACA CAT TAG AAC C | - | 2 µM | Reller et al. JCM (2013) [35] |
| PmPlasp4 rev | *P. malariae* | Plasp4 gene | GTA GGA TAT AAA GCA TAC ACA AAG TG | - | 2 µM | Reller et al. JCM (2013) [35] |
| PmPlasp4 probe | *P. malariae* | Plasp4 gene | ATC TAG TAA TGG CTC C | YakimaYellow-BHQ1 | 1.25 µM | Reller et al. JCM (2013) [35] |
| PoRBP2 fwd | *P. ovale* | Rpb2 gene | CCA CAG ATA AGA AGT CTC AAG TAC GAT ATT | - | 2 µM | Miller et al PLoS NTD (2015) [36] |
| PoRBP2 rev | *P. ovale* | Rpb2 gene | TTG GAG CAC TTT TGT TTG CAA | - | 2 µM | Miller et al PLoS NTD (2015) [36] |
| PoRBP2 probe | *P. ovale* | Rpb2 gene | TGA ATT GCT AAG CGA TAT C | TexasRed-BHQ2 | 1.25 µM | Miller et al PLoS NTD (2015) [36] |
| Pv18S fwd | *P. vivax* | 18S rDNA | GAC TAG GCT TTG GAT GAA AGA TTT TA | - | 2 µM | Cnops et al. CMI (2011) [33] |
| Pspp18S rev  (used by Pv and Pk) | *Plasmodium spp* | 18S rDNA | AAC CCA AAG ACT TTG ATT TCT CAT AA | - | 2 µM | Cnops et al. CMI (2011) [33] |
| Pv18S probe | *P. vivax* | 18S rDNA | GAA TTT TCT CTT CGG AGT TTA T | Cy5-BHQ2 | 1.25 µM | Cnops et al. CMI (2011) [33] |
| Pk18S fwd | *P. knowlesi* | 18S rDNA | GAA AGA TTT TAA AAT AAG AG |  | 2 µM | new design |
| Pk18S probe | *P. knowlesi* | 18S rDNA | CTC TCC GGA GAT TAG AAC TCT TAG ATT GCT | Quasar705-BHQ3 | 0.75 µM | Divis et al. MALARIA J (2010) [37] |
