# Supplementary material for: Molecular monitoring of the diversity of human pathogenic malaria species in blood donations on Bioko Island, Equatorial Guinea
Source: Malar J. 2019 Jan 15;18:9. doi: 10.1186/s12936-019-2639-8 (PMC6332537; doi:10.1186/s12936-019-2639-8)
Supplement: Supplementary file 2 — Additional file 2. Identification of risk factors associated with a malaria positive blood donation. [file 12936_2019_2639_MOESM2_ESM.docx]

Additional file 2. Identification of risk factors associated with a malaria positive blood donation.

| **English translation of question** | **negative donors (% answered with yes)** | ***P. falciparum* positive donors (% answered with yes)** | **OR [95% CI]** | **P value** |
| --- | --- | --- | --- | --- |
| Have you donated blood in the last 2 months, or more than 3 (women) or 4 times (men) in the last year? | 4.3% (6/141) | 3.9% (2/51) | 0.9184 [0.1793-4.7031] | 0.9186 |
| Have you ever been rejected as a donor or recommended not to donate blood? | 2.0% (3/147) | 4.0% (2/50) | 2.0 [0.3244-12.3290] | 0.4551 |
| Are you in good health today? | 95.9% (139/145) | 100.0% (52/52) | 4.8925 [0.2708-88.3799] | 0.2822 |
| Have you eaten anything in the last 4 hours? | 59.6% (87/146) | 58.8% (30/51) | 0.9688 [0.5066-1.8526] | 0.9237 |
| After donating, you will carry out an activity that endangers you or others in case of dizziness or faintness (Driver, crane operator, etc.)? | 9.1% (13/143) | 8.3% (4/48) | 0.9091 [0.2817-2.9339] | 0.8733 |
| Any heart disease (heart attack, angina, arrhythmia) or high blood pressure? | 1.4% (2/147) | 3.8% (2/52) | 2.9 [0.3979-21.1342] | 0.2934 |
| Any bronchial or lung disease (tuberculosis or asthma), kidney disease or diabetes? | 0.7% (1/147) | 0.0% (0/52) | 0.9302 [0.0373-23.1908] | 0.9648 |
| Cancer, serious bleeding or some blood disease? | 0.0% (0/147) | 0.0% (0/51) | 2.8641 [0.0561-146.2126] | 0.6000 |
| Any disease of the nervous system, seizures, fainting or epilepsy? | 0.0% (0/147) | 0.0% (0/52) | 2.8095 [0.0550-143.4017] | 0.6067 |
| Any disease of the digestive system, blood or metabolism? | 1.4% (2/146) | 0.0% (0/51) | 0.5612 [0.0265-11.8852] | 0.7107 |
| Any sexually transmitted disease, for example syphilis or gonorrhea? | 2.1% (3/146) | 2.0% (1/51) | 0.9533 [0.0969-9.3766] | 0.9673 |
| Hepatitis (you may also know it as "yellow fever"), Diabetes (high blood sugar), or some other disease chronicle? | 0.7% (1/145) | 0.0% (0/52) | 0.9175 [0.0368-22.8755] | 0.9581 |
| Have you been to the dentist last week? | 0.7% (1/145) | 0.0% (0/52) | 0.9175 [0.0368-22.8755] | 0.9581 |
| Have you taken any medication: antibiotic, aspirin, anti-inflammatory (ibuprofen) or other medications within the last week? | 13.8% (20/145) | 21.2% (11/52) | 1.6768 [0.7415-3.7918] | 0.2144 |
| Have you had a cold, flu, diarrhea or any other infection within the last three weeks? | 9.0% (13/145) | 11.5% (6/52) | 1.3244 [0.4757-3.6872] | 0.5907 |
| Have you had malaria and / or fever within the last three weeks? | 0.7% (1/146) | 9.6% (5/52) | 15.4255 [1.7575-135.3893] | 0.0136 |
| Have you been ill or have you received any treatment or medication within the last four months? | 6.8% (10/147) | 5.8% (3/52) | 0.8388 [0.2217-3.1741] | 0.7957 |
| Have you received any injection, vaccination or surgery within the last four months? | 3.5% (5/144) | 1.9% (1/52) | 0.5451 [0.0622-4.7785] | 0.5838 |
| Have you had typhoid within the last four months? | 4.2% (6/143) | 1.9% (1/52) | 0.4477 [0.0526-3.8103] | 0.4620 |
| Have you had sex outside of marriage within the last three months? | 9.1% (13/143) | 13.5% (7/52) | 1.5556 [0.5842-4.1422] | 0.3766 |
| Have you maintained risky relationships or a change of partner within the last three months? | 3.5% (5/144) | 7.8% (4/51) | 2.366 [0.6098-9.1793] | 0.2131 |
| Have you or your sexual partner received any blood transfusion within the last 6 months? | 0.7% (1/147) | 0.0% (0/52) | 0.9302 [0.0373-23.1908] | 0.9648 |
| Has a tattoo, body piercing or acupuncture treatment been done within the last year? | 2.0% (3/147) | 0.0% (0/52) | 0.3932 [0.0200-7.7414] | 0.5393 |
| Have you lived with a patient with hepatitis or been exposed to hepatitis within the last year? | 0.0% (0/146) | 0.0% (0/52) | 2.7905 [0.0547-142.4327] | 0.6090 |
| Have you been pregnant in the last 6 months or are you pregnant? | 0.0% (0/18) | 0.0% (0/2) | 7.4 [0.1182-463.1206] | 0.3429 |
| Are you breastfeeding? | 0.0% (0/18) | 0.0% (0/2) | 7.4 [0.1182-463.1206] | 0.3429 |
